# Sensory stimulation for upper limb amputations modulates adaptability of cortical large-scale systems and combination of somatosensory and visual inputs

**DOI:** 10.1038/s41598-022-24368-2

**Published:** 2022-11-28

**Authors:** Keqin Ding, Yunru Chen, Rohit Bose, Luke E. Osborn, Andrei Dragomir, Nitish V. Thakor

**Affiliations:** 1 Department of Biomedical Engineering, Johns Hopkins School of Medicine, Baltimore, MD USA; 2 Department of Bioengineering, University of Pittsburgh, Pittsburgh, PA USA; 3 Research and Exploratory Development Department, Johns Hopkins University Applied Physics Laboratory, Laurel, MD USA; 4 The N.1 Institute for Health, National University of Singapore, Singapore, Singapore; 5 Department of Biomedical Engineering, University of Houston, Houston, TX USA; 6 Department of Electrical and Computer Engineering, Johns Hopkins University, Baltimore, MD USA

**Keywords:** Biomedical engineering, Neuroscience

## Abstract

Touch-like phantom limb sensations can be elicited through targeted transcutaneous electrical nerve stimulation (tTENS) in individuals with upper limb amputation. The corresponding impact of sensory stimulation on cortical activity remains an open question. Brain network research shows that sensorimotor cortical activity is supported by dynamic changes in functional connections between relevant brain regions. These groups of interconnected regions are functional modules whose architecture enables specialized function and related neural processing supporting individual task needs. Using electroencephalographic (EEG) signals to analyze modular functional connectivity, we investigated changes in the modular architecture of cortical large-scale systems when participants with upper limb amputations performed phantom hand movements before, during, and after they received tTENS. We discovered that tTENS substantially decreased the flexibility of the default mode network (DMN). Furthermore, we found increased interconnectivity (measured by a graph theoretic integration metric) between the DMN, the somatomotor network (SMN) and the visual network (VN) in the individual with extensive tTENS experience. While for individuals with less tTENS experience, we found increased integration between DMN and the attention network. Our results provide insights into how sensory stimulation promotes cortical processing of combined somatosensory and visual inputs and help develop future tools to evaluate sensory combination for individuals with amputations.

## Introduction

For individuals with an upper limb amputation, the lack of afferent somatosensory inputs has been reported as one of the main reasons for decreased prosthesis usage^1^. Recent studies developed sensory stimulation strategies and showed promising results in reinstating touch-like sensations to these individuals^2–5^. The somatosensory information that was provided through stimulation improved individual’s performance in object manipulation and prosthesis control, demonstrating the importance of somatosensory input in the context of upper limb amputation. It is important to note that humans interact with the environment through multiple sensory inputs. Therefore, recent studies developed multisensory stimulation paradigms using combined visual and somatosensory inputs and showed improved sensory perception and prosthesis embodiment^6–8^. However, the cortical underpinnings of enhanced sensory perception and motor performance have not been commensurately investigated. Most literature demonstrates the benefits of sensory stimulation through behavioral and functional assessments such as questionnaires, phantom limb telescoping measurements, and proprioceptive drift measurements^6,9,10^. Consequently, understanding the cortical processing of sensory stimulation is crucial, because this knowledge can inform future design of effective stimulation paradigms.

Previous work collected electroencephalographic (EEG) recordings when individuals with amputations received sensory stimulation^5,11–14^. These studies showed evoked responses^11–14^ and increased activation in the central and centro-parietal regions of EEG signals^5^. These results validated that sensory stimulation elicited activity in the sensorimotor regions of the brain. Meanwhile, network neuroscience methods have allowed researchers to model cortical activity not as mere activation in the cortex, but as complex and intertwined functional connections between different cortical regions. This approach is suitable to study dynamic activity in the brain such as sensorimotor processing^15,16^. In this context, our previous work used dynamic functional connectivity and found that sensory stimulation to individuals with upper limb amputations led to increased functional connectivity between somatosensory, motor, and higher-level processing regions^17,18^.

Under the brain network paradigm, previous research involving individuals with upper limb amputations investigated changes in functional connections among cortical large-scale systems such as the somatomotor network (SMN) and the default mode network (DMN)^19^. These large-scale systems are typically established based on functional engagement of different regions^20^. As the brain processes internal and external information, cortical regions form several specific cognitive functional networks^21,22^. Among common large-scale systems, the DMN has been at the center of substantial research, owing to the conventional thought that the DMN facilitates spontaneous cognitive and stimulus-independent processes^23^. However, its functional role has not been completely elucidated yet. Recent work proposed that the DMN plays a role in contextualizing external sensory information with internal processes^24^. Furthermore, studies suggested that neural activity within the DMN may relate to brain activity in other cognitive systems, including those that are active during a task^25^. In this context, investigating cortical functions from the perspective of large-scale systems allows us to understand how changes resulting from external sensory information occur in the brain.

**Figure 1:**
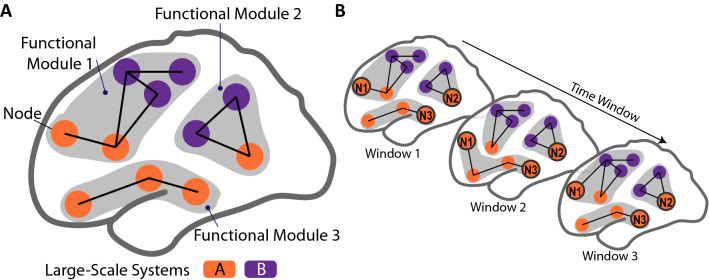
Schematic of functional modules and large-scale systems. (**A**) Each orange or purple circle represents a node (brain region). All orange nodes are part of large-scale system A and all purple nodes large-scale system B. Based on the functional connections and modularity, these nodes form three functional modules (shaded gray regions). (**B**) In a dynamic modular network, modules and connections may differ over time windows, as a result of adapting to different task demands. Node N1 switches which functional module it belongs to at each time window. This means node N1 has high *flexibility*. Node N2, part of large-scale system A (orange), is more connected to nodes in large-scale system B (purple) throughout the three time windows. This means node N2 has high interaction with nodes in a different large-scale system (high *integration*). In contrast, node N3 is more connected to nodes in large-scale system A. This means node N3 has high interaction with nodes in its own large-scale system (high *recruitment*).

It has often been suggested that in response to changes in external sensory information or environment, human brain function adapts to ensure the desired behavior^26^. As an individual with upper limb amputation performs a daily task, the most common external sensory information entails somatosensory information elicited via sensory stimulation, as well as visual information present in the environment. Previous studies showed that the reintroduced sensory information helped with fulfilling task requirements such as controlling a prosthesis or identifying object compliance^5,12^. At the cortical level, as we carry out different daily tasks, some brain regions (or nodes, in the functional connectivity formalism) dynamically change their interaction patterns with other regions to adapt to different task demands (Fig. 1A). This property, termed adaptability, is quantified by the *flexibility* metric and reflected as dynamic changes in groups of strongly connected brain regions, defined as functional modules. Modularity has been highlighted as a key characteristic of brain networks^27,28^. Understanding modules and adaptability is crucial when investigating brain functions because they provide insights into how information transmission is carried out in the brain^29^. Past studies on novel task practice and skill acquisition showed that brain *flexibility* initially increased and then decreased during a learning process^26^. In addition, cortical regions associated with higher-level processing functions, defined as cognitive activity that is more complex than primary sensory processing^30,31^, showed increased connectivity during early practice^32^.

In this study, we investigated three questions about the influence of sensory stimulation via targeted transcutaneous electrical nerve stimulation (tTENS) on large-scale systems and functional modules in the cortex (Fig. 1A). Does sensory stimulation influence the modular architecture of large-scale systems? What are the changes in the inter-connections between large-scale systems? Are the observed changes similar across individual participants? Addressing these questions could help develop assessment tools to evaluate how well an individual with upper limb amputations incorporates sensory stimulation.

To address these questions, we recorded EEG from individuals with upper limb amputations, each performing hand movements before, during, and after sensory stimulation that elicited sensations in their phantom hand. We also recruited intact-limb individuals for the same experiment. We investigated how dynamic modular networks characterized changes in interaction between large-scale systems and functional modules in response to sensory stimulation. We used graph theoretic metrics such as *flexibility*, *integration*, and *recruitment*, which quantify 1) changes among functional modules (*flexibility*) and 2) interactions within and across different large-scale systems (*integration* and *recruitment*) (Fig. 1B)^26,33^. We hypothesized that the presence of sensory stimulation would decrease the *flexibility* of the large-scale systems. This would indicate that executing a motor movement with sensory stimulation would not need as much functional adaptability and suggest that sensory stimulation could facilitate interaction between large-scale systems. In particular, based on recent work that suggested DMN involving in processing external sensory information, we expected that *flexibility* of DMN would decrease. For individuals with little experience with tTENS, we expected that systems with higher-level processing functions (e.g. the attention network) would be more active over the duration of the task.

## Results

Three participants with upper limb amputations (Table 1), with tTENS experience ranging from none to over 1.5 years, participated in this study. Three intact-limb participants (3 male, age range: 20–21) also participated. We first applied tTENS to each individual and performed sensory mapping to determine stimulation sites that elicited phantom sensations in participants with upper limb amputations and referred sensations in intact-limb participants. Stimulation targeted underlying peripheral nerves and could elicit median, ulnar, and radial regions of the phantom hand (see Methods for details).

**Figure 2:**
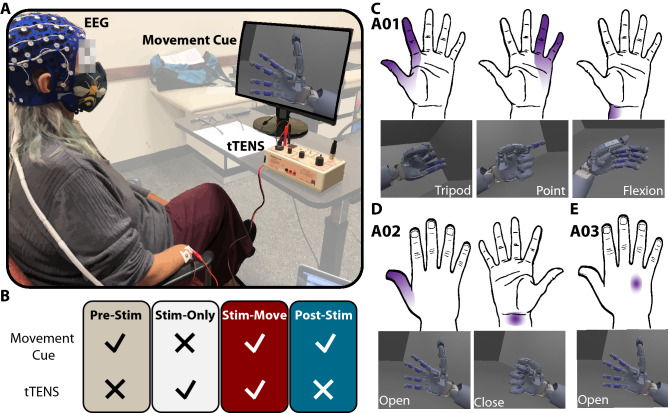
Experimental paradigm. (**A**) During the EEG experiment, the participant wore a 64-channel EEG cap and sat in front of a computer screen on which a visual cue of a hand movement was shown. The participant attempted the hand movement with or without tTENS. (**B**) The experiment consisted of four conditions: Pre-Stim, Stim-Only, Stim-Move, and Post-Stim. (**C**)–(**E**) Each participant with upper limb amputations selected different hand movements and stimulation regions. (**C**) Participant A01 associated median sensation with tripod, ulnar sensation with point, and wrist sensation with wrist flexion. (**D**) A02 associated median sensation with hand open, and wrist sensation with hand close. (**E**) A03 associated sensation on the back of the hand with hand open.

**Table 1:** Participant demographics.

Participant	Age (years)	Sex	Amputation	Time of amputation	tTENS experience
A01	29	Male	Left, transhumeral; right, transradial	2010	>1.5 years
A02	50	Male	Right, transradial	2017	5–10 days
A03	54	Female	Right, transradial; left, digits	2017	No experience

After sensory mapping, and prior to EEG recording, we asked each participant to associate tTENS elicited sensations with hand movements that were displayed on the computer screen (Fig. 2A). Participant A01 received stimulation on his left residual limb. He associated median sensation with tripod movement, ulnar sensation with index point movement, and wrist sensation with wrist flexion movement (Fig. 2C). Participant A02 received stimulation on his right residual limb. He associated median sensation with hand open, and wrist sensation with hand close (Fig. 2D). Participant A03 received stimulation on her right residual limb. She associated radial sensation on the back of her hand with hand open movement (Fig. 2E). For the group of intact limb participants, referred sensation was elicited in the intact hand when stimulating around the volar side of the wrist. The EEG experiment consisted of four conditions in the order of Pre-Stim, Stim-Only, Stim-Move, and Post-Stim (Fig. 2B). Each condition consisted of a visual movement cue, sensory stimulation, or simultaneous visual cue and stimulation. Each hand region was activated separately, based on the visual movement cue presented.

### Experience with sensory stimulation influences the change in modular architecture

We first investigated whether each individual’s history with sensory stimulation influenced change in modular architecture. Participant A01 had experience with tTENS for over 1.5 years (i.e., experienced with tTENS), while participant A02 had lesser experience (i.e., rudimentary experience with tTENS) and participant A03 had no experience with tTENS prior to our study (i.e., naive to tTENS) (Table 1).

**Figure 3:**
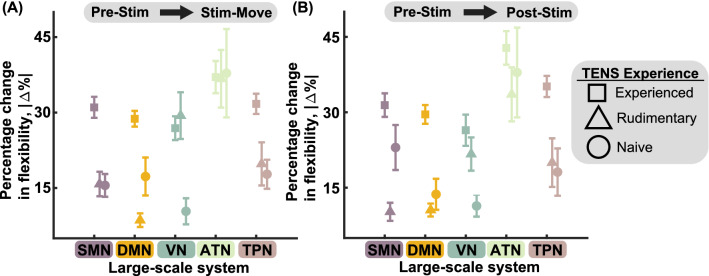
Percentage change in system-level *flexibility* for each level of tTENS experience. (**A**) Comparing Pre-Stim with Stim-Move, the percentage changes in *flexibility* of SMN, DMN, ATN, and TPN were the greatest for the participant with ample tTENS experience (A01). For all large-scale systems, the percentage change in *flexibility* was greater for the participant with rudimentary tTENS experience (A02) than that for the participant with no tTENS experience (A03). (**B**) Comparing Pre-Stim with Post-Stim, the percentage change in *flexibility* was the greatest in A01. For SMN, VN, and TPN, the percentage change was greater for A02 then for A03. SMN—Somatomotor network, DMN—Default mode network, VN—Visual network, ATN—Attention network, TPN—Task positive network. All error bars represent the standard error of the mean. Number of trials (n) for each point—Experienced: n = 30. Rudimentary: n = 20. Naive: n = 10.

To this end, we first localized the EEG data to cortical level activity using the low resolution brain electromagnetic tomography (LORETA) source localization algorithm^34^. We then allocated the localized areas into five predetermined large-scale systems: SMN, DMN, visual network (VN), attention network (ATN), and task-positive network (TPN) (see Methods and Supplementary Table S1). We calculated the *flexibility* metric to quantify the changes in modular architecture in response to sensory stimulation. More specifically, *flexibility* quantifies how often functional module assignment changes across the duration of a trial (2 seconds in this experiment). We then calculated the magnitude of percentage change in *flexibility* of each large-scale system between Pre-Stim and Stim-Move conditions (Fig. 3A), as well as Pre-Stim and Post-Stim conditions (Fig. 3B). We considered the experience with tTENS as an ordinal variable (i.e., experienced = 1, rudimentary = 2, naive = 3). We found that for all SMN, DMN, VN, and TPN, there was a negative correlation between the magnitude of percentage change in *flexibility* and the experience with tTENS of each participant (Table 2). The negative correlation was observed with 95% confidence.

These results suggest that the more experience an individual has with sensory stimulation via tTENS, the more changes in the brain’s modular architecture are observed. Based on the dependencies we found between changes in *flexibility* and each individual’s experience with tTENS, we performed the following analyses separately for each individual with upper limb amputations.

**Table 2:** Correlation between the magnitude of change in *flexibility* and tTENS experience.

Comparison	Statistical Measurement	System				
		SMN	DMN	VN	ATN	TPN
Pre-Stim versus Stim-Move	Correlation	−0.5321	−0.522	−0.2766	0.0082	−0.3939
	*p*-value	$1.2\times 10^{-5}$	$1.9\times 10^{-5}$	0.032	0.95	0.002
	95% CI	($-0.6925, -0.3217$)	($-0.6851, -0.3090$)	($-0.4957, -0.0244$)	($-0.2463, 0.2615$)	($-0.5889, -0.1555$)
Pre-Stim versus Post-Stim	Correlation	−0.3826	−0.6039	−0.3323	−0.1294	−0.4182
	*p*-value	0.003	$3.2\times 10^{-7}$	0.01	0.32	$8.8\times 10^{-4}$
	95% CI	($-0.5801, -0.1425$)	($-0.7438, -0.4134$)	($-0.5406, -0.0856$)	(−0.3711, 0.1280)	($-0.1555, -0.1838$)

### Sensory stimulation leads to decreased *flexibility* of the default mode network

**Figure 4:**
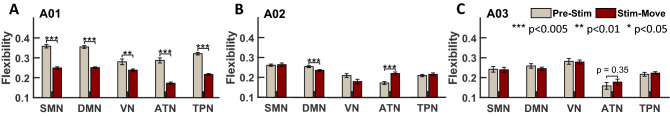
System level changes in *flexibility* during sensory stimulation (Stim-Move), compared to Pre-Stim for participants A01 (A), A02 (B), and A03 (C). (**A**) All large-scale systems showed decrease in *flexibility* for A01. (**B**) DMN showed decreased and ATN showed increased *flexibility* for A02. All error bars represent the standard error of the mean. A01: n = 30 each bar; A02: n = 20 each bar; A03: n = 10 each bar. $$^{***}p<0.005$$; $$^{**}p<0.01$$; $$^{*}p<0.05$$. See Supplementary Table S2 for statistical power analysis.

After evaluating the dependency between the magnitude of change in *flexibility* and experience with sensory stimulation, we investigated whether sensory stimulation via tTENS induced increase or decrease in *flexibility* for each individual. We first compared *flexibility* between Pre-Stim and Stim-Move conditions and found that for the experienced participant (A01), *flexibility* decreased for all five large-scale systems (SMN: $p=1.4\times 10^{-10}$, effect size $(\text {Cohen's }d_z) = 1.8$; DMN:$p=1.8\times 10^{-15}, \ d_z = 2.8$; VN: $p=0.009, \ d_z = 0.51$; ATN: $p=1.6\times 10^{-9}, \ d_z = 1.6$; TPN: $p=1.7\times 10^{-13}, \ d_z = 2.4$; Fig. 4A). For the participant with rudimentary experience with tTENS (A02), the changes in *flexibility* were statistically significant only for DMN and ATN (DMN: $p=0.001, \ d_z=0.86$; ATN: $p=0.0004, \ d_z=0.96$; Fig. 4B). In particular, we observed that *flexibility* of DMN decreased during Stim-Move for both participants A01 and A02. For the participant who had no experience with tTENS (A03), we did not observe significant statistical changes in *flexibility* between Pre-Stim and Stim-Move conditions. For A03, the greatest change was observed as increase in *flexibility* for ATN ($p=0.35,\ d_z=0.31$; Fig. 4C). The comparison between Pre-Stim and Stim-Move conditions for intact limb controls did not show statistical significance (Supplementary Fig. S1).

**Figure 5:**
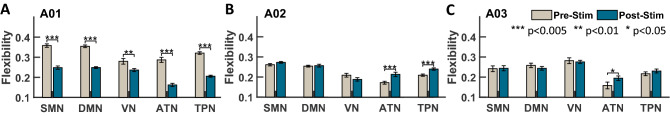
System level changes in *flexibility* after sensory stimulation (Post-Stim), compared to Pre-Stim for participants A01 (A), A02 (B), and A03 (C). (**A**) All large-scale systems showed decrease in *flexibility* for A01. (**B**) ATN and TPN showed increased *flexibility* for A02. (**C**) ATN showed increase in *flexibility* for A03. All error bars represent the standard error of the mean. A01: n = 30 each bar; A02: n = 20 each bar; A03: n = 10 each bar. $$^{***}p<0.005$$; $$^{**}p<0.01$$; $$^{*}p<0.05$$. See Supplementary Table S2 for statistical power analysis.

Comparing Pre-Stim and Post-Stim conditions, a similar decrease in *flexibility* of all five large-scale systems was observed in participant A01 (SMN: $p=1.5\times 10^{-10},\ d_z=1.8$; DMN:$p=1.6\times 10^{-12},\ d_z=2.1$; VN: $p=0.008,\ d_z=0.53$; ATN: $p=1.3\times 10^{-8},\ d_z=1.4$; TPN: $p=2.1\times 10^{-14},\ d_z=2.6$; Fig. 5A). Notably, for participants A02 and A03, ATN showed statistically significant increase in *flexibility* (A02: $p=0.002,\ d_z=0.83$; Fig. 5B; A03: $p=0.04,\ d_z=0.76$; Fig. 5C). TPN also showed increase in *flexibility* for A02 ($p=0.004,\ d_z=0.74$).

To summarize, we found decrease in *flexibility* of DMN across individuals with different levels of tTENS experience. Since the *flexibility* metric quantifies changes in functional module assignment, our observation suggests that performing a motor task required less amount of functional adaptability of DMN. In contrast, flexibility in ATN decreased during Stim-Move and Post-Stim for participant A01 and increased for participants A02 and A03. This result suggests the lesser role of attentional resources for the individual with extensive tTENS experience.

### Increased interaction among large-scale functional systems with increase in sensory stimulation experience

Our analysis of the *flexibility* metric showed that sensory stimulation changed the modular architecture of cortical large-scale systems, particularly in participants with previous tTENS experience. Here, we studied whether sensory stimulation influenced the interaction between these cortical large-scale systems. In particular, we focused on four systems—SMN, DMN, VN, and ATN—as they are most relevant to the sensory modalities and the task in our study. To this end, we computed *integration*, which quantifies the communication between nodes in different large-scale systems, and *recruitment*, which quantifies the communication between nodes within the same large-scale system (see Methods and Supplementary Methods for details on the computation of these metrics). These two metrics provide insight into whether nodes in the four systems are interacting more with nodes in their own system (high *recruitment*) or with nodes in other systems (high *integration*) during sensory stimulation (Stim-Move) and afterwards (Post-Stim). For each participant with upper limb amputations, we first examined overall changes in *integration* and *recruitment*. We then computed *integration* between each pair of the four large-scale systems and *recruitment* of each system.

**Figure 6:**
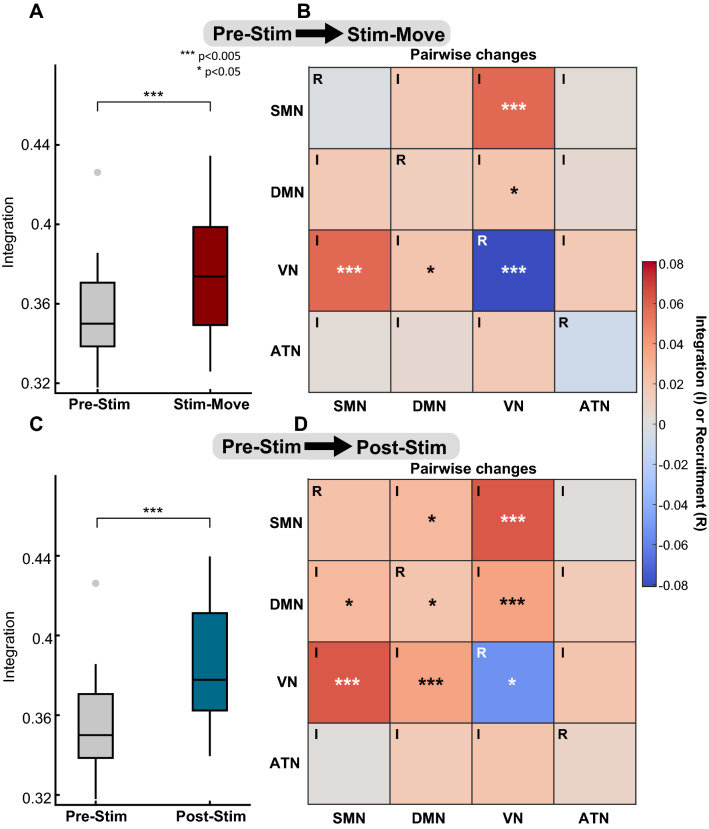
Changes in *integration* and *recruitment* of large-scale systems for participant A01. (**A**) Overall *integration* increased with statistical significance comparing Stim-Move with Pre-Stim. (**B**) Pairwise changes in *integration* and *recruitment* of the four large-scale systems (SMN, DMN, VN, and ATN) show statistically significance increase in *integration* between SMN and VN, and DMN and VN during Stim-Move. *Recruitment* of VN decreases. (**C**) Comparing Post-Stim with Pre-Stim, overall *integration* increased. (**D**) *Integration* increased between SMN and DMN, SMN and VN, and DMN and VN. *Recruitment* increased for DMN and decreased for VN. (**A**), (**C**) $n=30$ each box. (**B**), (**D**) Each square plots the average absolute change in *integration* (I) or *recruitment* (R) compared to Pre-Stim. Matrix plots are symmetric across the diagonal. All marked statistical significance was false discovery rate (FDR) corrected expect for VN-DMN *integration* in (**B**). See Supplementary Table S3 for statistical power analysis.

We first examined results from participant A01, who is experienced with tTENS. Comparing Stim-Move and Pre-Stim, we observed a statistically significant overall increase in *integration* of the four large-scale systems ($p=0.004,\ d_z=0.58$; Fig. 6A). We also observed a slight decrease in *recruitment* (Supplementary Fig. S2A). These results suggest that sensory stimulation can increase the interaction across different large-scale systems in the brain.

We then investigated pairwise *integration* and individual *recruitment* for each large-scale system. Pairwise *integration* focuses on two specific large-scale systems and provides details about which two systems are interacting more with each other when sensory stimulation is present. We found statistically significant increases in *integration* between two pairs of large-scale systems—SMN and VN ($p=0.0003,\ d_z=0.75$), DMN and VN ($p=0.02,\ d_z=0.45$) (Fig. 6B). For the visual network (VN), we observed a significant decrease in *recruitment* ($p=0.003,\ d_z=0.59$). These results suggest that during sensory stimulation, SMN and DMN are both interacting more with VN (increased *integration*), while VN is interacting less with itself (decreased *recruitment*).

In addition, we examined the interactions between large-scale systems based on functional connectivity strength of individual nodes, regardless of their modularity. To this end, we computed node-to-system density, which provides information about connectivity strength of nodes in one large-scale system with another large-scale system. Among nodes that showed statistically significant increase in *integration* (Fig. 6A), those in SMN had stronger connection density with VN, those in DMN tended to have stronger density with SMN, and those in VN tended to have stronger density with SMN (Supplementary Fig. S3A). These results further confirmed that the presence of sensory stimulation facilitated combination of somatosensory and visual inputs.

Finally, to understand whether sensory stimulation had after-effects beyond the stimulation period, we examined Post-Stim and Pre-Stim conditions. Overall, we found a significant increase in *integration* ($p=0.0008,\ d_z=0.67$; Fig. 6C). Pairwise *integration* changes showed increased *integration* between SMN and VN ($p=0.0009,\ d_z=0.45$), DMN and VN ($p=0.0003,\ d_z=0.77$), as well as SMN and DMN ($p=0.02,\ d_z=0.67$; Fig. 6D). DMN showed increased *recruitment* ($p=0.02,\ d_z=0.46$) and VN showed decreased *recruitment* ($p=0.01,\ d_z=0.48$). Additionally, for nodes that showed significant increase in *integration*, we observed those in SMN had stronger density with DMN, those in DMN had stronger density with SMN, and those in VN had stronger density with DMN (Supplementary Fig. S3B). These results in node-to-system density support our pairwise changes and indicate increased interaction between SMN and DMN, and VN and DMN.

### Increased interaction of the attention network for individuals with less or no tTENS experience

**Figure 7:**
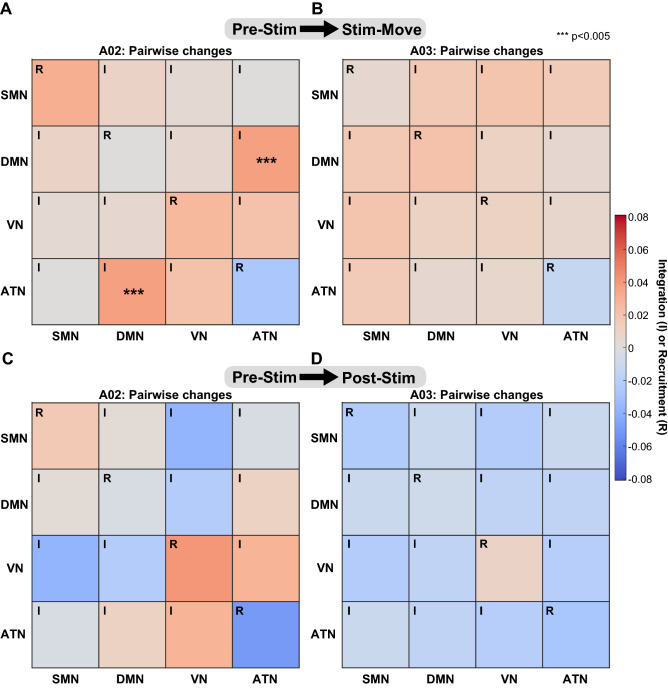
Changes in *integration* and *recruitment* of large-scale systems for participants A02 (A, C) and A03 (B, D). (**A**), (**B**) Comparing Stim-Move with Pre-Stim, pairwise *integration* generally increased. *Recruitment* of SMN, DMN, and VN increased and that of ATN decreased. (**A**) *Integration* between DMN and ATN increased with statistical significance for participant A02. (**C**), (**D**) Comparison between Post-Stim and Pre-Stim conditions. (**A**)–(**D**) Each square plots the average absolute change in *integration* (I) or *recruitment* (R) compared to Pre-Stim. All matrix plots are symmetric across the diagonal. See Supplementary Table S4 for statistical power analysis.

For participants A02 and A03, who are less experienced with tTENS, we observed less prominent changes in large-scale systems that are directly associated with a sensory modality (namely, SMN and VN). Overall *integration* and *recruitment* did not indicate considerable changes for these two participants (Supplementary Fig. S4A–H).

However, we found that the attention network (ATN) showed higher *integration* (Fig. 7), comparing Stim-Move with Pre-Stim. In particular, we found a statistically significant increase in *integration* between DMN and ATN during Stim-Move in participant A02 ($p=0.002,\ d_z = 0.79$; Fig. 7A and Supplementary Table S4). For nodes that showed statistically significant increase in *integration*, we found that those in DMN tended to have higher density with VN, and those in ATN tended to have higher density with DMN and VN (Supplementary Fig. S5). These node-to-system density results indicate increased interaction between DMN, ATN, and VN for participants with less tTENS experience. Results from A03 showed increase in pairwise *integration* between the four large-scale systems but were not statistically significant (Fig. 7B). In the Post-Stim condition, no statistically significant results were found in either A02 or A03 (Fig. 7C, D).

## Discussion

In this study, we aimed to investigate the influence of sensory stimulation on cortical large-scale systems and functional modules. We verified the hypothesis that dynamic modular networks reveal changes in interaction between large-scale systems and functional modules in response to tTENS. Our work provides insight into understanding the cortical processing of combined somatosensory and visual inputs while individuals with upper limb amputations performed phantom hand movements. While subjective or behavioral outcomes from participants with upper limb amputations can provide evidence for the benefits of sensory stimulation, detailed understanding of the cortical impacts is necessary for future design of optimal stimulation strategies and training regimens. Here, our work bridges the gap between the vast behavioral research on tactile sensations for individuals with upper limb amputations and the less explored aspect of cortical activity in response to sensory stimulation.

**Figure 8:**
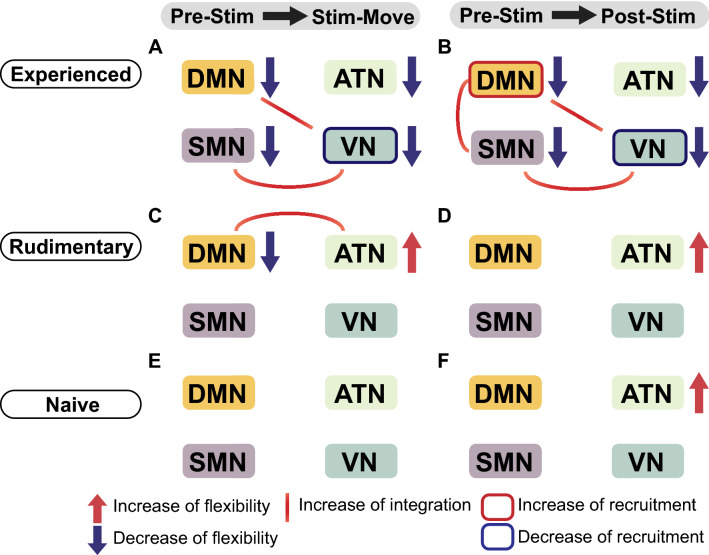
Summary schematic of statistically significant changes in *flexibility*, *integration*, and *recruitment* for participants with upper limb amputations. (**A**) For A01 who had extended experience with tTENS, comparison between Stim-Move and Pre-Stim shows decreased *flexibility* of all four large-scale systems, increased *integration* between SMN and VN, and between DMN and VN. VN shows decreased *recruitment*. (**B**) Comparison between Post-Stim and Pre-Stim for the experienced participant shows decreased *flexibility* of all four large-scale systems, increased *integration* between SMN, DMN, and VN. *Recruitment* of VN decreases. (**C**) For A02 who had limited experience with tTENS, comparison between Stim-Move and Pre-Stim shows decreased *flexibility* of DMN, increased *flexibility* of ATN, and increased *integration* between DMN and ATN. (**D**) Comparing Post-Stim with Pre-Stim for the less experienced (rudimentary), ATN shows increased *flexibility*. (**E**)–(**F**) For A03 who had no experience with tTENS (naive), comparison between Post-Stim and Pre-Stim shows increased *flexibility* of ATN.

In particular, we found that *flexibility* of DMN decreased in participants A01 (experienced) and A02 (rudimentary) when they performed phantom hand movement and received tTENS (Stim-Move condition, Fig. 8A, C). In the Post-Stim condition, *flexibility* of ATN increased in A02 and A03 (Fig. 8D, F). Our analysis on *integration* and *recruitment* showed two noticeable trends of changes: (1) increased *integration* between SMN, VN, and DMN for participant A01 who is experienced with tTENS (Fig. 8A, B), (2) increased *integration* between ATN and DMN for A02 who is less experienced with tTENS (Fig. 8C, D). These findings collectively suggest that sensory stimulation can facilitate cortical processing of combined somatosensory and visual inputs. Furthermore, the influences that tTENS has on the dynamic modular brain network likely differ based on each individual’s experience with tTENS.

### Enhanced activity between DMN, SMN, and VN in tTENS-experienced individual

Our results showed that *flexibility* of all large-scale systems decreased substantially in participant A01 when he made phantom hand movements with sensory stimulation (Fig. 4A). *Flexibility* quantifies how often functional modules change throughout the duration of a task, in order to adapt to task demands. Therefore, the overall decrease in *flexibility* suggests that with sensory stimulation, completing a motor task required less amount of functional adaptability for participant A01. The participant had over one and a half years of experience with sensory stimulation. Intriguingly, he also selected hand movements that required more precise finger control (i.e. index point and tripod) as opposed to merely hand open or close, which were selected by individuals with less tTENS experience (A02 and A03). The higher complexity of hand movements could be related to our observations. Previous studies on skill learning suggest that *flexibility* is desirable for large-scale systems in the brain to support new behavior and it decreases in the late stage of a learning process, when requirements of functional adaptability reduce^26,35^. In this regard, it is plausible that our observed decrease in *flexibility* indicates that the motor task of making a hand movement became less challenging and more “intuitive” to complete for participant A01.

While *flexibility* provides insights into whether functional modules change dynamically, *integration* and *recruitment* focus on the interaction within and between different large-scale systems, namely SMN, DMN, VN, and ATN in this study. Our results from participant A01 show that VN interacts more with SMN and DMN and less with itself during sensory stimulation (Fig. 6B). Higher *integration* between two large-scale systems indicates that these two systems are interacting with each other more often. Therefore, when participant A01 made phantom hand movements during sensory stimulation, the interaction between SMN and VN, and between DMN and VN increased. Further results on node-to-system density indicated stronger connectivity was formed between SMN and VN, as well as DMN and SMN (Supplementary Fig. S3A). It is important to note that SMN, DMN, and VN all showed decreased *flexibility* (Fig. 4A). Together, decreased *flexibility* and increased *integration* suggest that SMN, DMN, and VN form a more stable interaction pattern during sensory stimulation. Since our experiment includes two sensory inputs, namely somatosensory and visual, our findings suggest the presence of sensory stimulation facilitates cortical processing of combined somatosensory and visual inputs in participant A01.

Intriguingly, our results from participant A01 suggest that DMN is actively interacting with large-scale systems that are directly associated with a sensory modality. *Integration* between DMN and VN increased (Fig. 6A) and DMN formed higher connectivity density with SMN (Supplementary Fig. S3A). Functionally, DMN has long been associated with being active during “intrinsic” cognitive processes—spontaneous brain activity without external stimulation^36,37^. Other studies have shown that the DMN is situated at the top of the cortical sensory processing hierarchy, active when combining and processing information across different sensory modalities^38^. Therefore, the DMN combines incoming external information with internal knowledge to form a contextualized model^24^. In light of these recent interpretations of the DMN, our results suggest the DMN is active in processing the combined somatosensory and visual inputs to facilitate phantom hand grips (Fig. 2C).

The Post-Stim condition was used to explore the short term effects of sensory stimulation. Similar to our results in Stim-Move, *flexibility* of all large-scale systems also decreased for participant A01 (Fig. 5A), suggesting a persistent influence of sensory stimulation. Interestingly, *integration* in Post-Stim is even higher (Fig. 6C, D). In particular, increased *integration* between SMN and VN, as well as between DMN and VN persisted. Further, DMN is found to be more active during Post-Stim. There was statistically significant increase in *integration* between SMN and DMN compared to Pre-Stim (Fig. 6D). Additional results on node-to-system density also reveal that SMN and VN both tend to establish stronger density with DMN (Supplementary Fig. S3B). These observations are consistent with our prior work that enhanced activation of sensorimotor regions persisted in Post-Stim^5^. Here, our findings on sustained *integration* suggest that sensory stimulation could have short-term influences on the large-scale systems in the brain. The interaction between somatosensory and visual networks persisted even after the stimulation ended.

### Increased communication between DMN and ATN in individuals with less experienced with tTENS

Our results suggest that for participants with amputations who had little to no experience with tTENS, large-scale systems that are associated with higher-level processing (namely DMN and ATN) are more active. *Flexibility* of DMN decreased and *flexibility* of ATN increased significantly for participant A02 (Fig. 4B). Interestingly, DMN showed decrease in *flexibility*, similar to our observation in participant A01 (Fig. 4A), suggesting nodes in DMN are changing their functional modules less frequently when sensory stimulation was provided. The increased *flexibility* of ATN indicates the modular architecture of ATN shifted more frequently during sensory stimulation.

The significant increase in *integration* between DMN and ATN of participant A02 during Stim-Move (Fig. 7A) suggests that DMN and ATN show heightened interaction during sensory stimulation. In addition, DMN tends to establish stronger connection density with VN, and ATN with DMN and VN (Supplementary Fig. S5). These results suggest that, for participant A02, DMN is more actively interacting with ATN.

Large-scale systems that are directly associated with a sensory modality (SMN and VN) are less active in participants A02 and A03 during Stim-Move. Rather, both SMN and VN showed trends of increased *recruitment* during sensory stimulation, though these observations were not supported by significant statistical results. Increased *recruitment* of a system implies that the system tends to interact more with itself. Therefore, our results might suggest that SMN and VN are not as actively involved in interacting with other systems and that sensory combination is not as well facilitated for participants A02 and A03 as it is for participant A01. Overall, the increased activity of ATN with DMN suggests that higher-level processing is required for participants A02 and A03, who had less experience with tTENS. This interpretation is supported by a previous study that demonstrates brain networks that are associated with higher-level processing were only active during the early practice stage of a skill acquisition task^32^.

Our results for the Post-Stim condition also show that there was a statistically significant increase in *flexibility* of ATN for both A02 and A03 (Fig. 5B, C). This finding further suggests increased activity of ATN for these two individuals. We did not observe statistically significant changes in *integration* and *recruitment* during Post-Stim (Fig. 7C, D), similar to our intact limb participants (Supplementary Fig. S6). It may be because A03’s limited experience of tTENS was not beneficial to her in making a phantom hand movement during the Post-Stim condition and the influences of sensory stimulation to the dynamic modular networks were more transient. Further, sensory stimulation and feedback could have a learning period, during which individuals with upper limb amputation familiarize themselves with the sensations. Potential future work could track changes in *integration* and *recruitment* of large-scale systems as subjects become more experienced with tTENS.

### Implications for sensory stimulation

Our work shows that DMN is actively interacting with other systems for A01 and A02, while ATN is more active for A02 and A03. We postulate that each individual’s experience with tTENS can explain the differences we observe. For the tTENS-experienced individual, increased *integration* between SMN and VN were observed both during Stim-Move and in Post-Stim. Together with the increased activity of DMN, this could indicate that sensory stimulation facilitated the interaction between the two large-scale systems, each directly associated with a specific sensory modality (i.e., somatosensory and visual). Further, when such interaction was present, the task of performing a phantom hand movement showed improved outcome. Our previous work showed improved motor decoding accuracy after sensory stimulation^5^.

When participants were less experienced with tTENS, higher-level processing may be required in the brain to incorporate this reintroduced sensation. For participants A02 and A03, increased ATN activity suggests more processing is exerted between large-scale systems that are not directly associated with a sensory modality. Information on *recruitment* of VN suggests that there could be a process of honing in on the crucial requirements for completing a hand movement task. In particular, the tTENS-experienced individual showed decrease in *recruitment* of VN. These observations could indicate that the tTENS-experienced individual could focus on the task itself, rather than trying to use the presented information to do the task.

The differences we observed based on experience with sensory stimulation could also be related to the fidelity of phantom sensations provided and the complexity of hand movements each individual selected. Sensation fidelity was not directly studied, but it is likely that our participant with extensive tTENS experience (A01) perceived the phantom sensations better. With increased quality of the perceived sensations, the participant could then better associate the location of the sensation with him performing phantom hand movements. We can not yet infer the causality among tTENS experience, sensation fidelity, and movement complexity. Therefore, further research is needed to link these factors and cortical large-scale system interaction together.

It is important to note that while our experiment included somatosensory and visual inputs, the setup was different from previous work on multisensory stimulation, which focused more on providing tactile and visual feedback to improve behavioral and functional outcomes^6–8^. Regardless, the cortical processing of combined somatosensory and visual inputs need to be understood in the context of upper limb amputation. In our work and within the cognitive neuroscience framework, sensory combination constitutes the merging of multiple sensory signals to form an unambiguous percept^39^. In the context of sensory stimulation for upper limb prostheses, the added artificial somatosensory information helps an individual perceive the environment through multiple sensory inputs. Here, our results showed the combination of somatosensory and visual inputs was manifested in terms of dynamic modular networks in the cortex. In particular, the interaction pattern we observed between SMN, DMN and VN for our tTENS-experienced participant could be an indication of effective combination of multiple sensory inputs. Our work suggests the possibility of using dynamic modular networks to assess the cortical processing of combined sensory inputs. Future studies on cortical large-scale systems could include simultaneous somatosensory and visual feedback to further explore cortical responses with prosthesis usage.

In conclusion, investigating the influence of sensory stimulation on large-scale systems could serve as a tool for mechanistic exploration of sensory combination. Insights into how well a stimulation approach promotes high interaction of somatosensory and visual networks could be important for designing effective stimulation strategies for improved prosthesis perception and control.

## Methods

This study was approved by the Johns Hopkins Medicine (JHM) Institutional Review Board. All study experiments were performed in accordance with the relevant guidelines and regulations of JHM and the ethical standards of the Declaration of Helsinki. Each participant provided their written informed consent to participate in this study. Additionally, a written informed consent was obtained from each participant to have their images taken for an online open access publication. No known neurological disorders were found or reported in any of the participants.

### Experiment

Monophasic square wave pulses were provided to each participant by a beryllium copper probe connected to an isolated current stimulator (DS3, Digitimer Ltd, UK). Sensory mapping was performed to identify stimulation sites that elicited phantom sensations in participants with upper limb amputations and referred sensations in intact-limb participants. Areas of the residual limb were scanned with the beryllium copper probe, targeting underlying nerve bundles, namely the median, ulnar, and radial nerves. During sensory mapping, an outline of a hand was used for each individual to report the activation regions in the phantom hand. The corresponding stimulation site was marked. Amplitudes of 0.5–2.6 mA, frequencies of 2–4 Hz and pulse widths of 1–5 ms were used as stimulation parameters. Details of the aforementioned sensory mapping procedures and parameters were adopted from our published work^5,17,40^.

Sixty-four-channel EEG signals were collected during the experiment with silver/silver chloride electrodes sampling at a frequency of 500 Hz using a SynAmps2 system (Compumedics Neuroscan, Charlotte, NC, USA). A modified version of the Virtual Integration Environment (VIE) (Johns Hopkins University Applied Physics Lab, Laurel, MD, USA) was used to provide visual cues of the selected hand movements^41^ (Fig. 2A). During the Pre-Stim condition, each participant was provided with a visual cue of selected movements and attempted to perform hand movements with no sensory stimulation was provided. Participants with upper limb amputations attempted to perform the movement shown by the visual cue with their phantom hand and the intact-limb controls performed the movement with their intact hand. During the Stim-Only condition, each participant was provided with tTENS but no visual cue of movement and did not perform the movement grips. During the Stim-Move condition, each participant was provided with both tTENS on the associated sites previously determined in sensory mapping as well as visual cue of movements. During the Post-Stim condition, each participant performed the movement again only with a visual cue and without tTENS.

In each condition, one trial refers to the duration in which the visual cue and/or sensory stimulation was presented for 2 seconds followed by a 4-second delay with ±25% jitter. For A01, each movement cue was presented 10 times in the Pre-Stim condition and 20 times in all other conditions. For all other participants, each movement cue was presented 20 times in each condition. Sensory stimulation during the experiment used monophasic square waves with a frequency of 45–50 Hz, a pulse width of 1–2 ms, and an amplitude of 0.5–2.6 mA based on our previous work^5,17^.

### EEG signal processing

The sixty-four-channel EEG recordings were processed using the EEGLAB toolbox in MATLAB^42^. A fifth-order Butterworth filter was applied with a low frequency cutoff of 0.3 Hz and high frequency cutoff of 50 Hz. A notch filter at 60 Hz was then applied on the bandpass-filtered signals to remove power line artifacts. Independent component analysis was used to further reduce artifacts caused by eye, muscle, or other movements during data collection. The data was segmented into epochs starting from 1 second before the start of each trial and ending at 2 seconds after stimulus onset. The 2 seconds from the stimulus start point (including tTENS and/or visual stimuli) were used for analysis. We focused on the alpha (8–13 Hz) frequency band and the Pre-Stim, Stim-Move and Post-Stim conditions.

We localized the preprocessed EEG data to cortical level activity using exact low resolution brain electromagnetic tomography (eLORETA)^34^, then further divided the source-localized EEG data into 80 regions of interest (ROIs, also referred to as nodes) based on the automated anatomical labeling (AAL) atlas^43^. Most ROIs were assigned to one of the five predetermined large-scale functional systems based on the cortical area each ROI belongs to and its functional role with respect to task performance. The five large-scale functional systems that are relevant to our study are: SMN, DMN, VN, TPN, and ATN. The remaining non-categorized ROIs were labelled as “Others” (Supplementary Table S1, Fig. S7A)^44–48^.

### Dynamic modular network construction

We used a sliding window (300 ms window length with 50% overlap) on the source-localized data for each trial to compute undirected functional connectivity. The functional connectivity metric was the weighted phase lag index (WPLI), which calculates the phase leads and lags normalized by the imaginary component of the instantaneous phase difference between two time series signals^49^ [Supplementary Methods Eq. (1)]. For each trial, a total of 12 time windows formed a weighted multilayer graph, which models the dynamic cortical response to sensory stimulation as functional interdependencies between nodes (Supplementary Fig. S8).

To obtain the functional modules, we used the generalized Louvain (GenLouvain) community detection algorithm [Supplementary Methods Eqs. (2) and (3)]^50,51^. Since this algorithm is non-deterministic, we repeated community detection 500 times for each trial in each condition. All metrics computed were averaged over the 500 repetitions.

### Dynamic modular network metrics

For each repetition of the community detection algorithm, we computed three main dynamic modular network metrics. The first metric is *flexibility* (F), which computes the ratio of the number of times a node changes its functional module (S), to the total number of temporal windows (T), or $F=\frac{S}{T}$^52^. Nodes that have low *flexibility* change their corresponding functional modules less frequently than those with high *flexibility* throughout a trial during a task^26,53^ (Supplementary Fig. S7B). The second metric is *integration*, defined as the probability that a node co-occurs in detected functional modules with nodes from different large-scale systems. We computed (a) overall *integration*, which considers the average *integration* of nodes in all large-scale systems, and (b) pairwise *integration*, which considers *integration* between two specific pairs of large-scale systems [Supplementary Methods Eqs. (4)–(6)]^33^. The third metric is *recruitment*, which computes the probability that a node co-occurs in functional modules with nodes from the same large-scale system, thereby describing the connections between nodes within the same large-scale system [Supplementary Methods Eqs. (7), (8)]^33^. The *recruitment* and *integration* metrics shed light on interaction between and within large-scale systems. We also calculated node-to-system density, which computes the average connectivity strength between a node in one system and all nodes in another system [Supplementary Methods Eq. (9)]^54^.

### Statistical analysis

Pearson’s correlation coefficients were calculated between tTENS experience and magnitude of percentage change in *flexibility*. Two-tailed paired t-tests were used to compare: 1) Pre-Stim and Stim-Move conditions and 2) Pre-Stim and Post-Stim conditions. Normality of the dynamic modular network metrics were confirmed using an Anderson-Darling test. The metrics for one condition were averaged for participants with repeated trials. False discovery rate (FDR) with statistical significance level $\alpha = 0.05$ was performed after each paired t-test. Cohen’s $d_z$ was used to represent effect sizes [Eq. (1)]^55^.

1 $$\begin{aligned} d_z = \frac{\mu _{(X_1 - X_2)}}{SD_{(X_1 - X_2)}},\ X_1 \text { and } X_2 \text { represent the same metric from two different conditions.} \end{aligned}$$

Unless otherwise stated, statistical comparison results were all FDR corrected. For all statistical testings that resulted in $p<0.05$, post hoc statistical power analysis was conducted using G*Power^56^.

## Supplementary Information

Supplementary Information.

## Data Availability

The datasets generated and/or analyzed during the current study are available from the corresponding author on reasonable request.
