# Supplementary material for: Sensory stimulation for upper limb amputations modulates adaptability of cortical large-scale systems and combination of somatosensory and visual inputs
Source: Sci Rep. 2022 Nov 28;12:20467. doi: 10.1038/s41598-022-24368-2 (PMC9705529; doi:10.1038/s41598-022-24368-2)
Supplement: Supplementary file 1 — Supplementary Information. [file 41598_2022_24368_MOESM1_ESM.pdf]

Sensory stimulation for upper limb amputations modulates adaptability of cortical large-scale systems and combination of somatosensory and visual inputs

Keqin Ding[1,*], Yunru Chen[1], Rohit Bose[2], Luke E. Osborn[3], Andrei Dragomir[4,5], Nitish V. Thakor[1,6]
[1] Department of Biomedical Engineering, Johns Hopkins School of Medicine, Baltimore, MD, USA
[2] Department of Bioengineering, University of Pittsburgh, Pittsburgh, PA, USA
[3] Research and Exploratory Development Department, Johns Hopkins University Applied Physics Laboratory, Laurel, MD, USA
[4] The N.1 Institute for Health, National University of Singapore, Singapore
[5] Department of Biomedical Engineering, University of Houston, Houston, TX, USA
[6] Department of Electrical and Computer Engineering, Johns Hopkins University, Baltimore, MD, USA
[*] kding3@jhu.edu

Supplementary Methods

Table S1. ROI names, acronyms, and corresponding systems[1–5]

System	Node Name [a]	Acronym [a]	System	Node Name	Acronym
Somatomotor (SMN)	Precentral Gyrus	PreCG	VN	Middle occipital	MOG
	Inferior frontral gyrus, opercular	IFGoperc		Inferior occipital	IOG
	Supplementary motor area	SMA	Attention (ATN)	Inferior frontal gyrus, triangular	IFGtriang
	Postcentral	PoCG		Temporal pole: supeior temporal gyrus	TPOsup
	Paracentral lobule	PCL		Temporal pole: middle temporal gyrus	TPOmid
Default Mode (DMN)	Superior frontal gyrus, dorsolateral	SFGdor	Task Positive (TPN)	Superior frontal gyrus, orbital	ORBsup
	Superior frontal gyrus, medial	SFGmed		Middle frontal	MFG
	Superior frontal gyrus, medial orbital	ORBsupmed		Middle frontal gyrus, orbital	ORBmid
	Anterior cingulate and paracingulate gyri	ACG		Inferior frontal gyrus, orbital	ORBinf
	Median cingulate and paracingulate gyri	DCG		Rolandic operculum	ROL
	Posterior cingulate gyrus	PCG		Insula	INS
	Supramarginal gyrus	SMG		Superior parietal gyrus	SPG
	Angualr gyrus	ANG		Inferior parietal, but supramarginal and angular	IPL
	Precuneus	PCUN	Others	Olfactory	OLF
	Heshl gyrus	HES		Gyrus rectus	REC
	Superior temporal gyrus	STG		Parahippocampal gyrus	PHG
	Middle temporal gyrus	MTG		Amyglada	AMYG
Visual (VN)	Calcarine fissure and surrounding cortex	CAL		Lingual gyrus	LING
	Cuneus	CUN		Fusiform gyrus	FFG
	Superior occipital gyrus	SOG		Inferior temporal gyrus	ITG

[a] Node and acronym names are the same for the left and right hemispheres.

Functional connectivity and dynamic modular network estimation

$$\Phi = \frac{|E\{|\Im\{X\}|sgn(\Im\{X\})\}|}{E\{|\Im\{X\}|} \quad (1)$$

Φ is WPLI, X is the cross-spectrum and $\Im\{X\}$ is the imaginary part of the cross-spectrum X.

The GenLouvain algorithm estimates a multi-layer modularity matrix and assumes homogeneous ordinal coupling and a Newman-Girvan null model on each layer[6]. The GenLouvain community detection algorithm was iterated until the difference between modularity ($Q_{multislice}$) of the current and the last community partitions is smaller than 10^{-6}.

$$\mathrm{Q_{multislice}} = \frac{1}{2\mu}\sum_{\mathrm{ijsr}}[(\mathrm{A_{ijs}} - \gamma_{\mathrm{s}}\mathrm{V_{ijs}})\delta_{\mathrm{sr}} + \delta_{\mathrm{ij}}\mathrm{C_{jsr}}]\delta(\mathrm{g_{is}}, \mathrm{g_{jr}}) \quad (2)$$

i and j represent nodes, s and r represent time windows.

For community detection to converge, generalized modularity across slices was calculated as $Q_{multislice}$, where $\mu = \frac{1}{2}\sum_{jr}\kappa_{jr}$ is the total edge weight in pne multilayer network. κ_{jr} is the strength of node j in window s calculated by $\kappa_{jr} = k_{jr} + c_{jr}$, where $k_{jr} = \sum_i A_{ijr}$, and $c_{jr} = \sum_s C_{jsr}$. A_{ijs} is the component in adjacency matrix A of window s, C_{jsr} is the connection strength of node j between window s and window r. g_{is} represents the community assignment of node i in time window s and g_{jr} represents the community assignment of node j in time window r[6,7].

$$V_{ijs} = \frac{k_{is}k_{js}}{2m_s} \quad (3)$$

V_{ijs} represents Newman-Girvan null model, where the total edge weight m_s is computed by $m_s = \frac{1}{2}\sum_{ij} A_{ijs}$, k_{is} is the strength of node i in window s, k_{js} is the strength of node j in window s. Equations 2–3 were implemented using a generalized Louvain method toolbox[8].

Dynamic modular network metrics

***Integration* and *Recruitment* metrics**

$$M_{ij} = \frac{1}{OT}\sum_{o=1}^{O}\sum_{t=1}^{T} a_{i,j}^{k,o}, \text{ where } a_{i,j}^{k,o} = \begin{cases} 1 & \text{if node } i \text{ and node } j \text{ co-occur in the same community} \\ 0 & \text{otherwise} \end{cases} \tag{4}$$

M_{ij} stands for functional module allegiance—the probability at which two nodes co-occur in the same functional module. O is the number of repetitions (500 in this study). T is the number of time windows (12 time windows in this study).

$$I_i^S = \frac{1}{N - n_S}\sum_{j \notin S} M_{ij} \tag{5}$$

I_i^S is the *integration* metric I of node i in large-scale system S, N is the total number of nodes in this analysis, and n_s is the total number of nodes in the system.

$$I_{S_y S_z} = \frac{1}{n_{S_y} n_{S_z}}\sum_{i \in S_y}\sum_{j \in S_z} M_{ij} \tag{6}$$

$I_{S_y S_z}$ is the pairwise *integration* between large-scale systems S_y and S_z.

$$R_i^S = \frac{1}{n_S}\sum_{j \in S} M_{ij} \tag{7}$$

R_i^S is the *recruitment* metric R of node i in network S.

$$R_S = \frac{1}{n_S^2}\sum_{i \in S}\sum_{j \in S} M_{ij} \tag{8}$$

R_S is the *recruitment* metric of an individual large-scale system S.

Calculation of node-to-system density

$$d_{i,v} = \frac{1}{N_v}\Sigma_{j \in v, i \in k, k \neq v}\, w_{ij} \tag{9}$$

where $d_{i,v}$ is the node-to-system density between node i, in system k, and system v. The connection w_{ij} is defined as the connection in the weighted phase lag index matrix between node i in system k and node j in system v. The sum is further normalized by the number of nodes in system v to output the node-to-system density for node i in system k to system v (adapted from[9]).

References

1. Vossel, S., Geng, J. J. & Fink, G. R. Dorsal and ventral attention systems. *The Neurosci.* **20**, 150–159, DOI: 10.1177/1073858413494269 (2013).

2. Dosenbach, N. U., Fair, D. A., Cohen, A. L., Schlaggar, B. L. & Petersen, S. E. A dual-networks architecture of top-down control. *Trends Cogn. Sci.* **12**, 99–105, DOI: 10.1016/j.tics.2008.01.001 (2008).

3. Higo, T., Mars, R. B., Boorman, E. D., Buch, E. R. & Rushworth, M. F. S. Distributed and causal influence of frontal operculum in task control. *Proc. Natl. Acad. Sci.* **108**, 4230–4235, DOI: 10.1073/pnas.1013361108 (2011).

4. Menon, V. & Uddin, L. Q. Saliency, switching, attention and control: a network model of insula function. *Brain Struct. Funct.* **214**, 655–667, DOI: 10.1007/s00429-010-0262-0 (2010).

5. Seghier, M. L. The angular gyrus: Multiple functions and multiple subdivisions. *The Neurosci.* **19**, 43–61, DOI: 10.1177/1073858412440596 (2012).

6. Mucha, P. J., Richardson, T., Macon, K., Porter, M. A. & Onnela, J.-P. Community structure in time-dependent, multiscale, and multiplex networks. *Science* **328**, 876–878, DOI: 10.1126/science.1184819 (2010).

7. Mattar, M. G., Cole, M. W., Thompson-Schill, S. L. & Bassett, D. S. A functional cartography of cognitive systems. *PLOS Comput. Biol.* **11**, e1004533, DOI: 10.1371/journal.pcbi.1004533 (2015).

8. Jeub, L. G. S., Bazzi, M., Jutla, I. S. & Mucha, P. J. A generalized louvain method for community detection implemented in MATLAB. https://github.com/GenLouvain/GenLouvain (2019).

9. Taya, F. *et al.* Fronto-Parietal Subnetworks Flexibility Compensates For Cognitive Decline Due To Mental Fatigue. *Hum. Brain Mapp.* **39**, 3528–3545, DOI: 10.1002/hbm.24192 (2018).

Supplementary Results

Table S2. Statistical power of *flexibility* metric for participants A01, A02, and A03[a]

Participant ID	Comparison	Large-scale system	Statistical Power
A01	Pre-Stim vs Stim-Move	SMN	> 0.99
		DMN	> 0.99
		VN	0.77
		ATN	> 0.99
		TPN	> 0.99
	Pre-Stim vs Post-Stim	SMN	> 0.99
		DMN	> 0.99
		VN	0.79
		ATN	> 0.99
		TPN	> 0.99
A02	Pre-Stim vs Stim-Move	DMN	0.95
		ATN	0.98
	Pre-Stim vs Post-Stim	ATN	0.94
		TPN	0.88
A03	Pre-Stim vs Post-Stim	ATN	0.58

[a] related to Fig. 4 and Fig. 5

Table S3. Statistical power of *integration* and *recruitment* metrics for participant A01[a]

Comparison	Metric (system if applicable)	Statistical Power
Pre-Stim vs Stim-Move	Overall *Integration*	0.87
	Pairwise *Integration* (SMN-VN)	0.98
	Pairwise *Integration* (DMN-VN)	0.66
	Recruitment (VN)	0.88
Pre-Stim vs Post-Stim	Overall *Integration*	0.95
	Pairwise *Integration* (SMN-DMN)	0.66
	Pairwise *Integration* (SMN-VN)	0.95
	Pairwise *Integration* (DMN-VN)	0.98
	Recruitment (DMN)	0.69
	Recruitment (VN)	0.71

[a] related to Fig. 6

Table S4. Statistical power of *integration* and *recruitment* metrics for participants A02 and A03[a]

Comparison	Metric (system if applicable)	Statistical Power
Pre-Stim vs Stim-Move	Pairwise *Integration* (DMN-ATN)	0.92

[a] related to Fig. 7

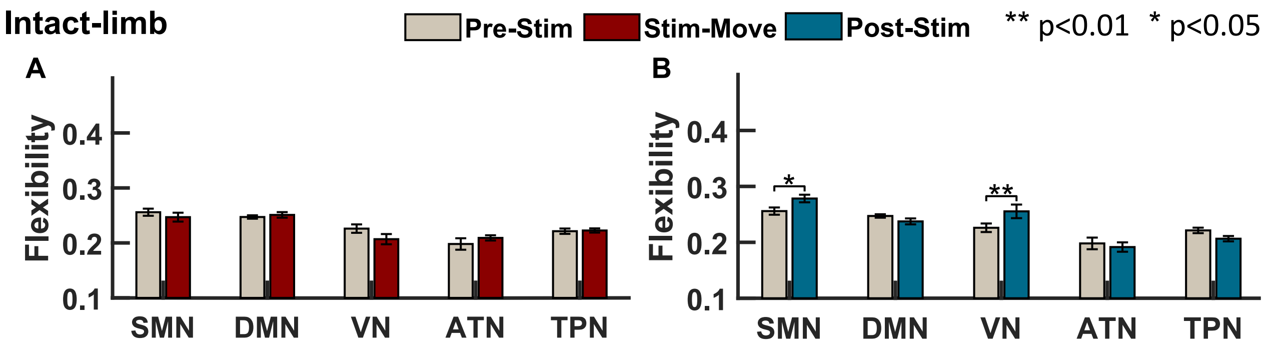

Figure S1. System level changes in *flexibility* for intact limb participants, comparing Stim-Move with Pre-Stim (**A**), and Post-Stim with Pre-Stim (**B**). (n=10 each box)

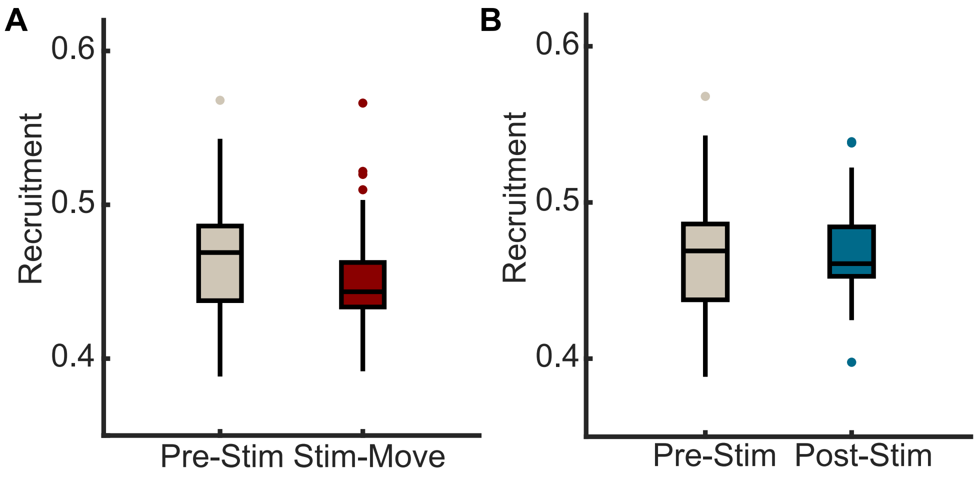

Figure S2. Overall change in *recruitment* for participant A01. **(A)** Comparing Stim-Move with Pre-Stim, *recruitment* shows a decreasing trend. **(B)** Same as (A), comparing Post-Stim with Pre-Stim. In both comparisons, overall *recruitment* considers nodes in all four large-scale systems: SMN, DMN, VN, and ATN. (n=30 each box)

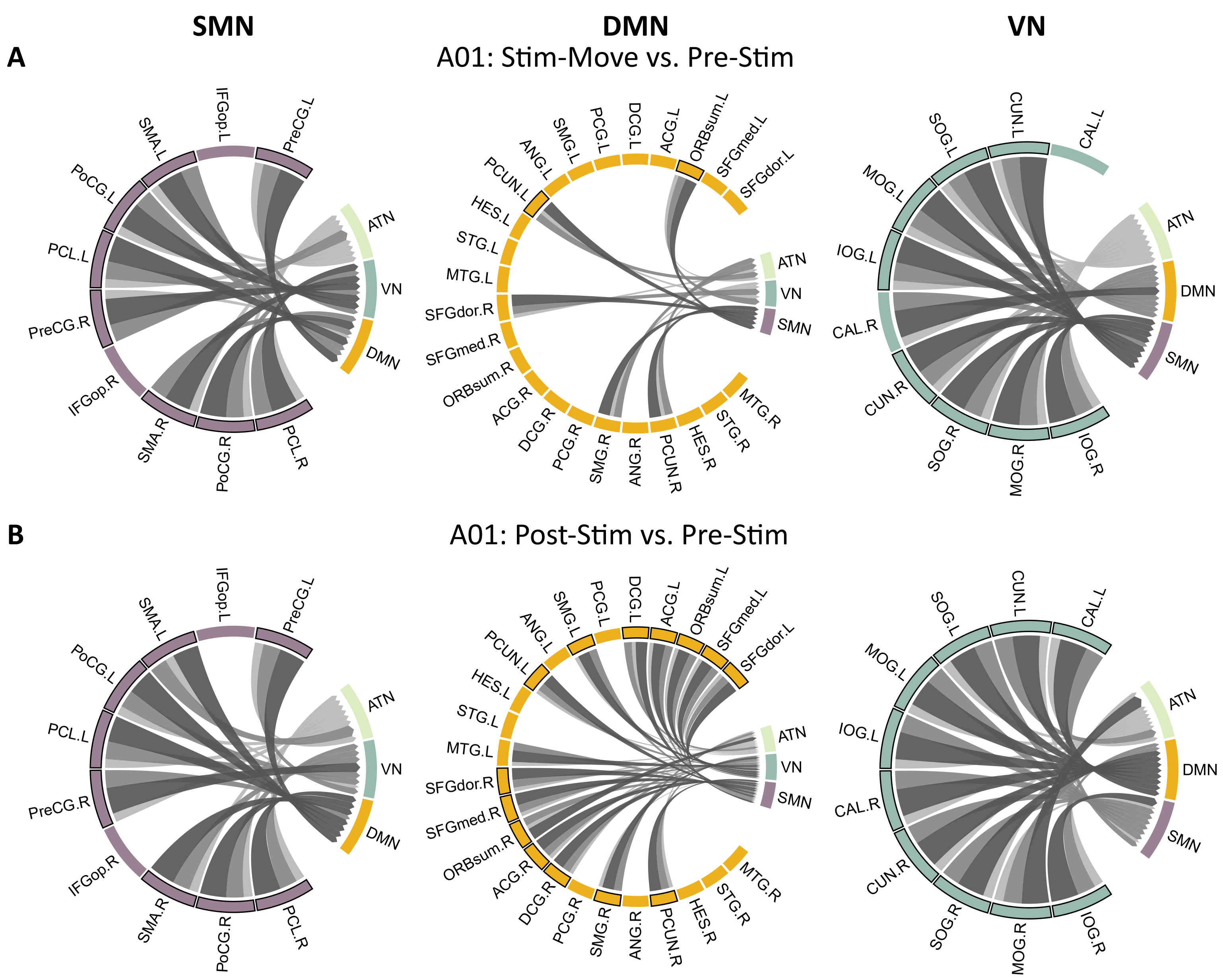

Figure S3. Node to system density for nodes showing increased *integration* for participant A01 in Stim-Move (**A**) and Post-Stim (**B**). In each circular plot, the left side represents nodes in the originating system and the right represents the receiving system names. For each node, we ranked the three node-to-system density values it has with the three systems on the right. The darkness of the connection bands represents the ranking, such that a darker shade means higher node-to-system density. Black borders around node names indicate statistical significance after FDR correction.

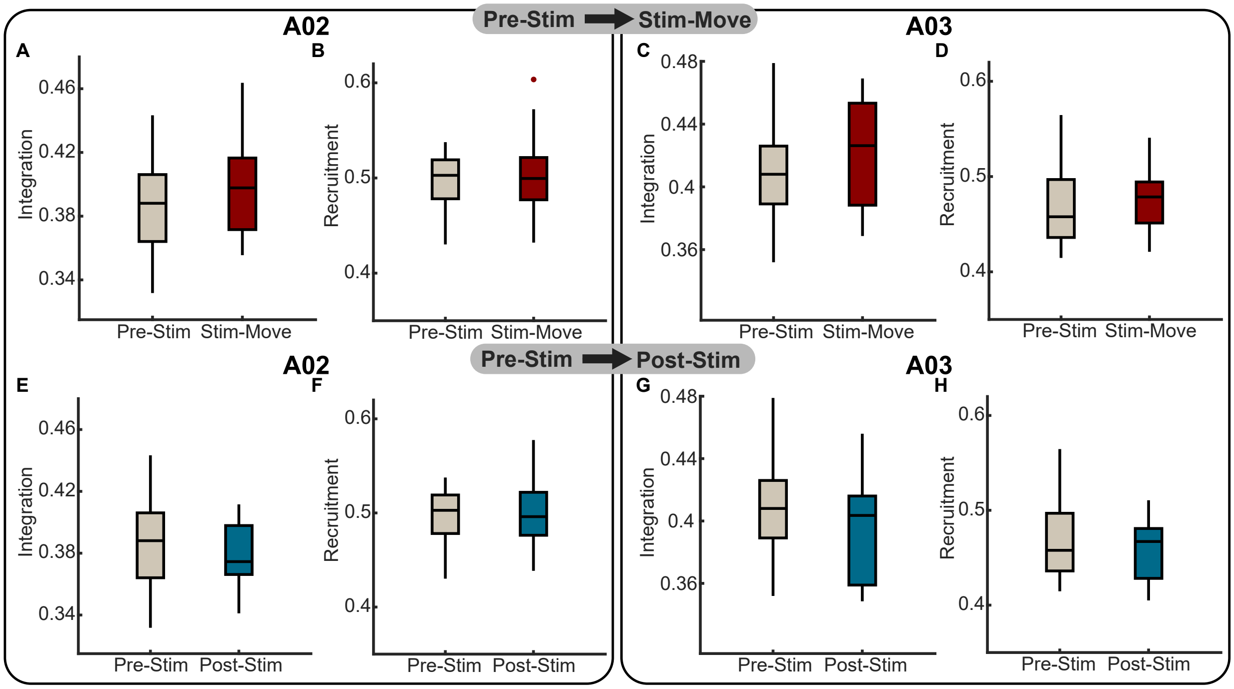

Figure S4. Overall change in *integration* and *recruitment* for participants A02 and A03, comparing Stim-Move with Pre-Stim **(A–D)**, and Post-Stim with Pre-Stim **(E–H)**. A02: n=20 each box; A03: n=10 each box.

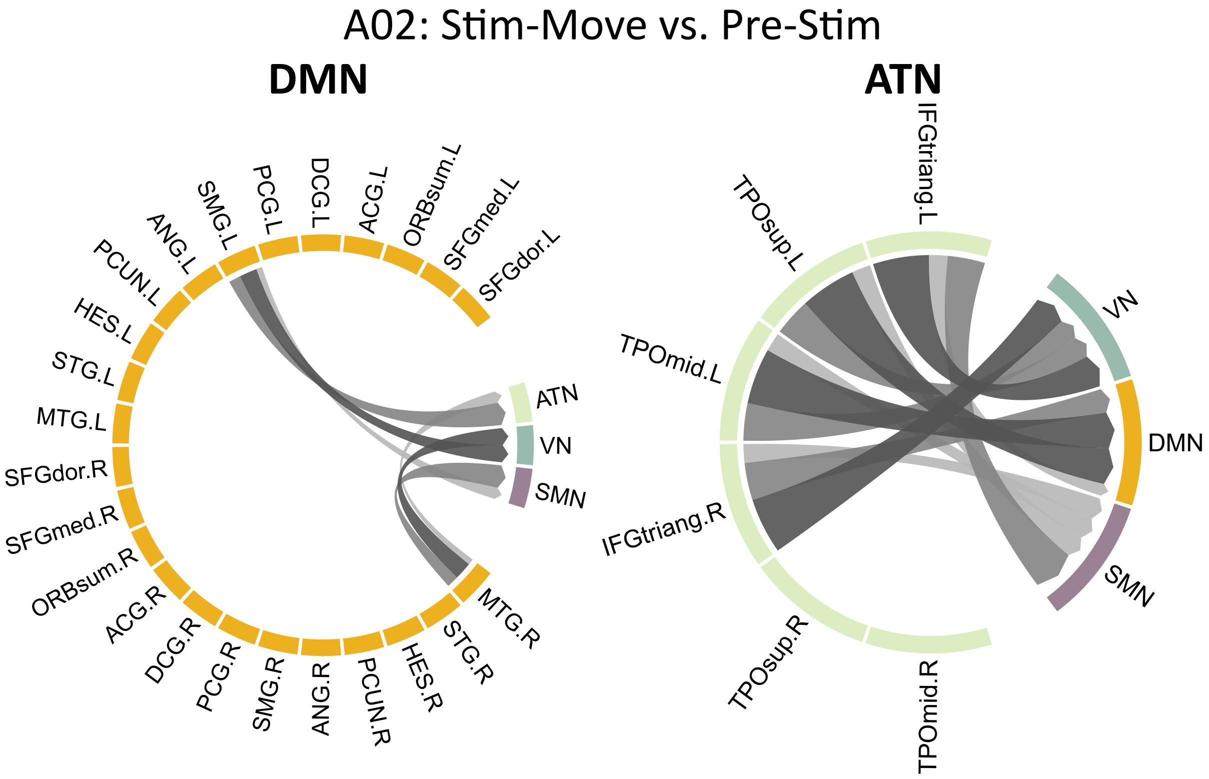

Figure S5. Node to system density for nodes showing increase in *integration* for participant A02 in Stim-Move. The left side of each circular plot represents nodes in the starting system and the right represents the receiving system names. In each circular plot, the left side represents nodes in the originating system and the right represents the receiving system names. For each node, we ranked the three node-to-system density values it has with the three systems on the right. The darkness of the connection bands represents the ranking, such that a darker shade means higher node-to-system density.

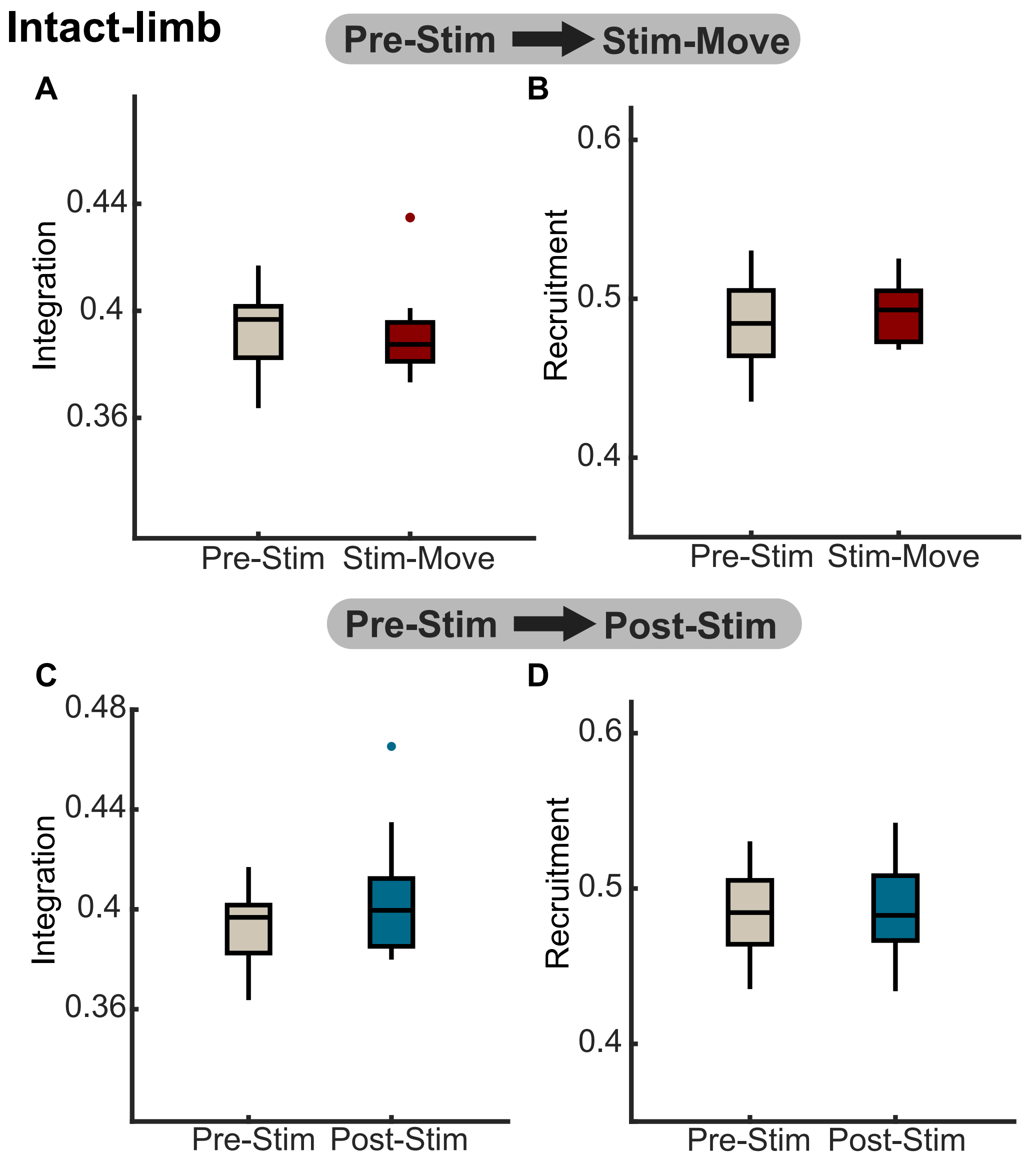

Figure S6. Large-scale system level changes of *integration* and *recruitment* for the three intact-limb participants. **(A, B)** Comparing Stim-Move with Pre-Stim, *integration* **(A)** and *recruitment* **(B)** do not show observable changes. **(C, D)** Same with (A, B), comparing Post-Stim with Pre-Stim. n=10 each box.

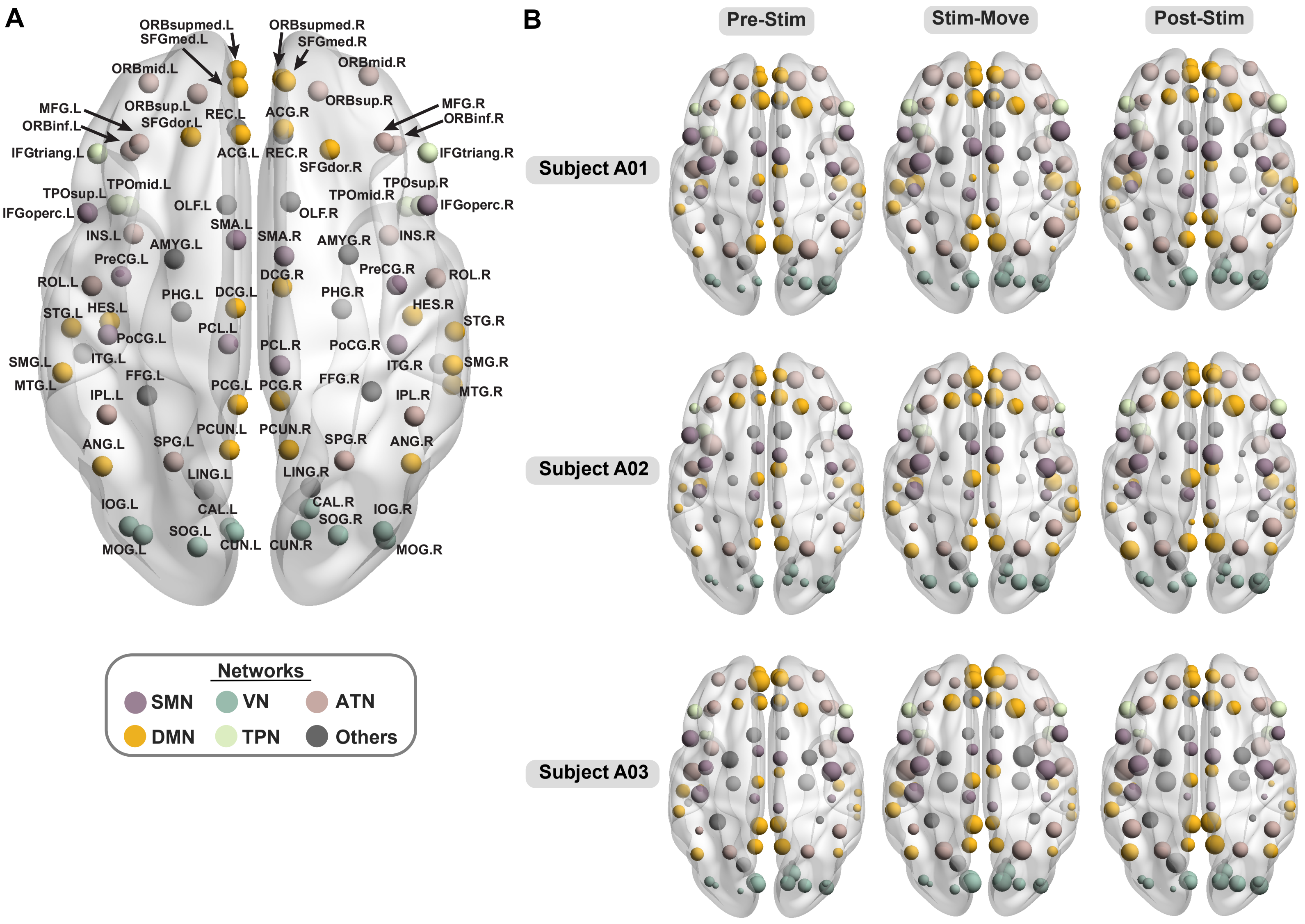

Figure S7. **(A)** Brain schematic of all 80 ROIs, color coded by the large-scale system. **(B)** Schematic of the *flexibility* metric for each participant in each condition. The size of the nodes represent the magnitude of *flexibility*.

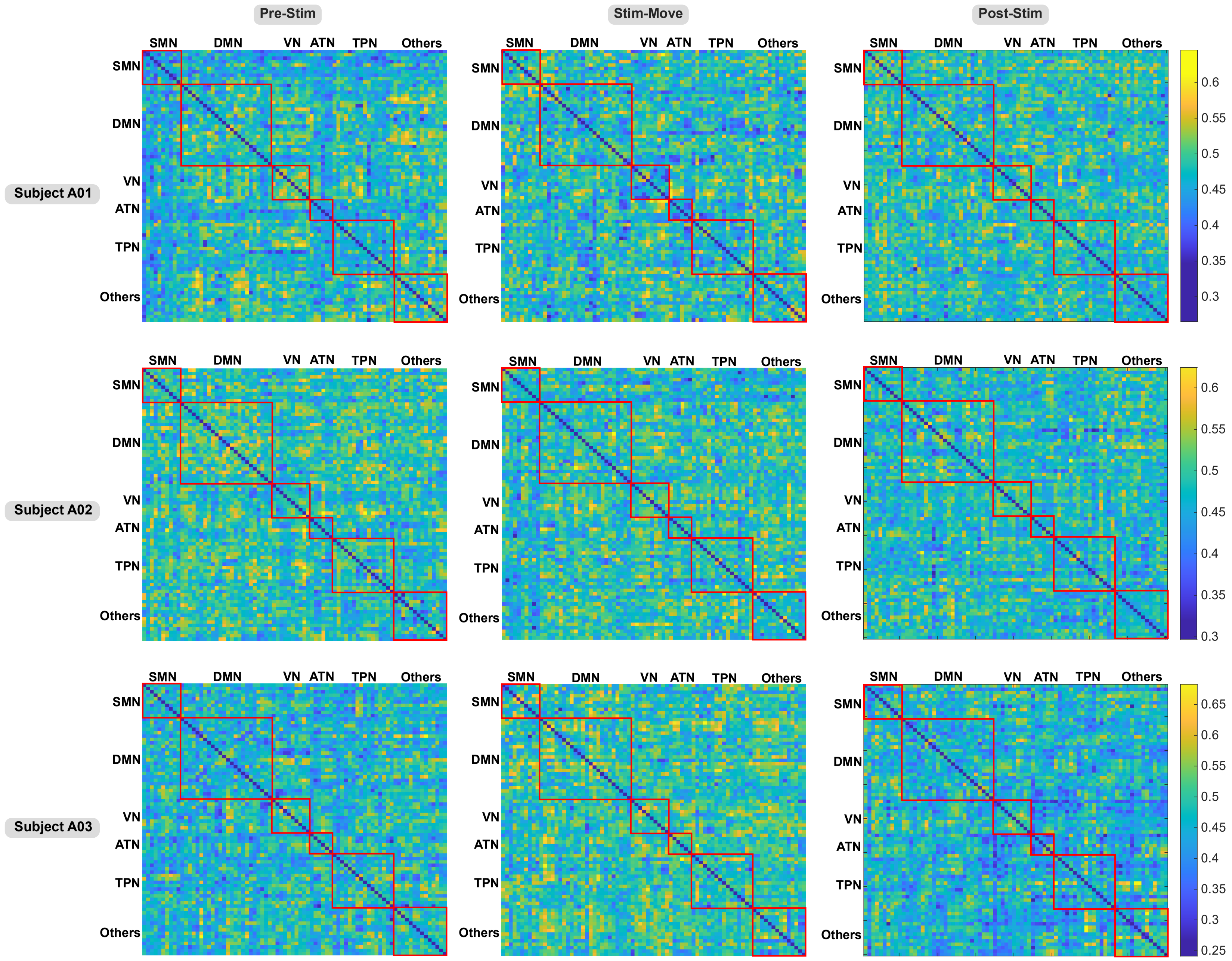

Figure S8. Grand average wPLI for each participant and condition. Red boxes represent large-scale systems.
